# Supplementary material for: Comparative Characterization of Key Volatile Compounds in Slow- and Fast-Growing Duck Raw Meat Based on Widely Targeted Metabolomics
Source: Foods. 2022 Dec 8;11(24):3975. doi: 10.3390/foods11243975 (PMC9778640; doi:10.3390/foods11243975)
Supplement: Supplementary file 1 [file foods-11-03975-s001.zip › Table S1 & Figure S1.pdf]

Table S1. Ingredient and nutrient levels of the commercial diets in ducks (1~65 d).

Item	1~21 d	22~65 d
Ingredients, %		
Corn	56.00	74.10
Soybean meal	27.50	18.40
Wheat meal	10.00	0.87
Fish meal	5.00	5.50
Limestone	1.00	0.70
Salt	0.24	0.24
DL-Methionine	0.16	0.09
Vitamin and trace mineral mix	0.10	0.10
Nutrient levels		
Apparent ME, KJ/ kg	12.34	12.97
CP, %	22.00	18.00
Lys, %	1.23	0.94
Met, %	0.52	0.40
Trp, %	0.29	0.23
Thr, %	0.91	0.76
Ca, %	0.87	0.75
Total P, %	0.41	0.39
Sulphur-amino acid, %	0.85	0.66

Note: Vitamin and trace mineral mix supplied the following per kilogram of total diet: Cu ($CuSO_4{\cdot}5H_2O$), 10 mg; Fe ($FeSO_4{\cdot}7H_2O$), 60 mg; Zn (ZnO), 60 mg; Mn ($MnSO_4{\cdot}H_2O$), 80 mg; Se ($NaSeO_3$), 0.3 mg; I (KI), 0.2 mg; choline chloride, 750 mg; vitamin A (retinyl acetate), 8,000 IU; vitaminD3 (Cholcalciferol), 3,000 IU; vitamin E (DL-a-tocopheryl acetate), 20 IU; vitamin K_3 (menadione sodium bisulphate), 2 mg; thiamin (thiamin mononitrate), 1.5 mg; riboflavin, 4 mg; pyridoxine hydrochloride, 3 mg; cobalamin, 0.02 mg; calcium-D-pantothenate, 10 mg; nicotinic acid, 50 mg; folic acid, 1 mg; and biotin, 0.15 mg. ME: Metabolic Energy. CP: Crude protein.

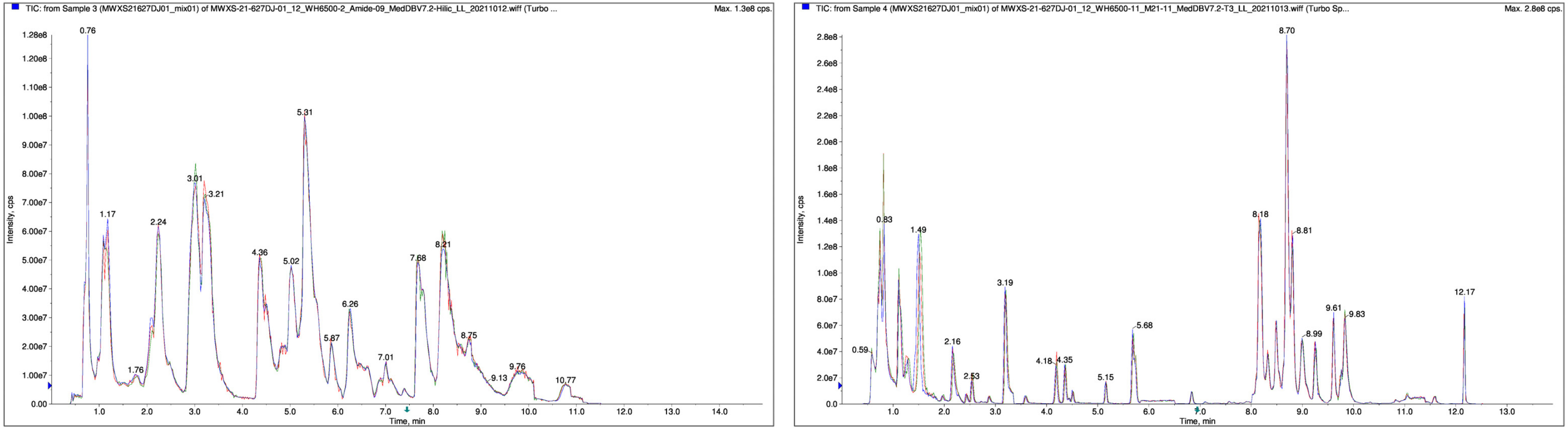

Figure S1: TIC overlap detected by essential spectrum of QC sample.
